# Relationships Between Emotional Stability, Psychosocial Mentoring Support and Career Resilience

**DOI:** 10.5964/ejop.v11i1.835

**Published:** 2015-02-27

**Authors:** Ridhi Arora, Santosh Rangnekar

**Affiliations:** a Department of Management Studies, Indian Institute of Technology Roorkee, Roorkee, Uttarakhand, India; Aalborg University, Aalborg, Denmark

**Keywords:** Big Five, emotional stability, mentoring, personality, career resilience

## Abstract

This study empirically investigates the mediating role of psychosocial mentoring support on emotional stability personality disposition and career resilience relationship. In addition, this research also focuses on estimating the interrelationship between emotional stability, psychosocial mentoring support and career resilience. The results show substantive direct relations between emotional stability and psychosocial mentoring as well as between emotional stability and career resilience. Psychosocial mentoring is also seen as a significant predictor of career resilience. Further, it mediates partially the relationship between emotional stability personality and career resilience. Future and practical implications of research have also been provided.

## Introduction

The topic of mentoring has grabbed the attention of academicians and management practitioners worldwide. Mentoring is regarded as the most popular form of developmental relationships, which stimulates the career advancement of employees (Bozionelos & Wang, 2006). Mentoring relationship exists between *mentor*, a senior experienced person and *protégé*, a junior experienced person in which psychosocial support of the mentor (friendship, unconditional acceptance and confirmation, counseling, role-modeling) is essential in promoting the protégé’s competence, self-efficacy and overall development (Kram, 1985; Shollen, Bland, Center, Finstad, & Taylor, 2014). Also, such type of mentoring has been demonstrated to be effective in enhancing persistence of the employees during the adverse times (Carson & Bedeian, 1994; Day & Allen, 2004; London, 1983). This extent to remain motivated despite the challenges and fluctuations in a given line of work/career is known as career resilience (Carson & Bedeian, 1994).

Managing career resilience represents a major challenge both for organizations and employees due to the effects of globalization, changing career trends and increased pace of technology. This has forced organizations to devise career-resilience building strategies such as mentoring activities for employees with varied personality traits. In this context, the “Big Five” or “OCEAN” model of personality provides the most appealing framework for understanding a wide range of behaviors in terms of five broad personality traits—openness, conscientiousness, extraversion, agreeableness and neuroticism (emotional stability) (Neff, Toothman, Bowmani, Fox Tree, & Walker, 2011). Of these Big Five traits, we are interested in studying emotional stability that ranges between two extremes—emotionally stable and neurotic—and is also one of the widely examined personality trait (Neff et al., 2011). The emotional stability personality trait has potential implications for regulating protégé’s varied range of emotions at all stages of the mentoring relationship (Ragins & Kram, 2007; Turban & Lee, 2007). For example, while high emotional stability indicates high self-assurance, low emotional stability is likely to impede progress at all the stages of the mentoring relationship, thereby, leading to dysfunctional mentoring relationships (Turban & Lee, 2007). Therefore, for handling such protégés, psychosocial support of the mentor is likely to be influential in motivating them to be resilient in every situation.

### Aim and Hypotheses Development

**Figure 1:**
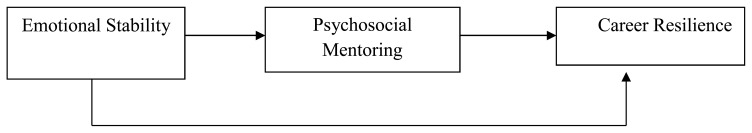
Proposed model.

In this study, we propose to examine the interrelationships between emotional stability, psychosocial mentoring and career resilience with a specific focus on investigating the mediating role of psychosocial mentoring on the relationship between emotional stability and career resilience. The proposed model of the study has been represented in Figure 1. We aim to propose these objectives based on social exchange theory (SET) and cognitive developmental theory. These theories postulate that social exchange relationships evolve when employers “take care of employees,” to productively influence work behavior and positive employee attitudes. Such social exchange relationships, like mentoring also help in the intellectual stimulation of the protégé through the provision of ample growth opportunities (Cropanzano & Mitchell, 2005; Rhodes, Spencer, Keller, Liang, & Noam, 2006). Thus, by investigating the mediating role of psychosocial mentoring on emotional stability and career resilience, our research theoretically and empirically expands our knowledge on mentoring and career resilience, as not many studies have been conducted in relation to mentoring with career-related variables, like career resilience (Arora & Rangnekar, 2014). The survey of existing literature shows that previous studies have investigated about how mentoring produces resilience in women, adolescents and medical students (Kao, Rogers, Spitzmüller, Lin, & Lin, 2014). However, limited studies have been found on mentoring and career resilience within the organizational context. Besides this, a plethora of studies on the mediating role of psychosocial mentoring support also highlights the significance of conducting this research.

### Emotional Stability

Emotional stability dimension is recognized as a significant predictor of job performance (Rothmann & Coetzer, 2003), and is also known for its key role in maintaining conducive workplace social interactions (Lee, Dougherty, & Turban, 2000). Low scorers on emotional stability are labeled as neurotic individuals, who have a tendency to display ineffective coping mechanisms, and also carry a hostile attitude as well as they are self-blaming in nature. They lack the ability to find constructive solution to a problem (Clutterbuck & Lane, 2004), and show their indecisiveness very often. Further, low emotional stability also suggests about an individual’s constant struggle with the feelings of insecurity and self-consciousness (Costa & McCrae, 1992a; Goldberg, 1993). Such people are prone to psychiatric problems. Thus, while lower scores on this factor experience a range of negative emotions, such as stress, anxiety, anger, embarrassment, disgust, guilt and fear (Rothmann & Coetzer, 2003), higher scores on this factor have a tendency to remain self-assured, calm and free from fluctuating and disturbing emotions.

### Career Resilience

In the past, various perspectives have been represented to guide the concept of resilience that includes both psychological and environmental perspectives (Bimrose & Hearne, 2012). Bryant (1995) has defined resilience as “whenever a change occurs in a person’s life, the individual traits and skills (e.g. time management) interact with both environmental as well as situational factors to produce a behavior that leads to the successful adaptation towards change.” Therefore, resilience is “an ability to absorb the high level of disruptive change while displaying minimal dysfunctional behavior” (Conner, 1992). Again according to Richardson (2002), resilience is “the motivational force within everyone that creates a drive to pursue wisdom, self-actualization, altruism and to be in harmony with the spiritual source of strength.”

One such form of resilience is career resilience that highlights “the ability to adapt to change even when the circumstances are discouraging (Bimrose & Hearne, 2012; London, 1997). According to Conner (1992), resilient people tend to enhance the total assimilation of the change impact. Thus, even though it may be difficult for them to accept any change during the change implementation process, they still remain engaged and regain their equilibrium faster. Later on Fourie and Van Vuuren (1998) stated that career resilient people are endowed with a great ability to adapt during changing circumstances. Similarly, Abu-Tineh (2011) in his research on 100 faculty members of Qatar University, explained career resilience as one’s ability to manage effectively one’s work life. According to him, members with high resilience are flexible, goal-driven, high in self-esteem, instilled with confidence, optimist, and are able to make timely changes and always provide new learning opportunities for implementation.

### Psychosocial Mentoring

Psychosocial mentoring support covers “those aspects of a relationship that enhance an individual’s sense of competence, identity and effectiveness in a professional role” (Kram, 1985, p. 32). Psychosocial mentoring includes various functions, such as mentors serving as *role-model;* conveying unconditional positive regard toward protégé through unconditional *acceptance and confirmation;* encouraging protégé to discuss his anxieties and fears without any hesitation and *counseling* him by informally interacting with him by becoming his *friend* (Kram, 1985; Noe, 1988). These functions promote the personal growth of the protégé with the aid of mentor’s emotional support and guidance (Chao, 1998). According to Simon, Perry, and Roff (2008), psychosocial mentoring functions operate at an interpersonal level, and represent a deeper and a more intense aspect of the mentoring relationships (Allen, Eby, Poteet, Lentz, & Lima, 2004). Such type of mentoring often evolves into a more emotional bond and a pleasurable positive interpersonal contact develops between the mentor and the protégé (Raabe & Beehr, 2003).

### Emotional Stability and Career Resilience

Individuals low on emotional stability (neuroticism) is characterized by instability, depression and also display a lack of personal insecurity. Such people have a tendency to remain hostile and impulsive because of which they often show lack of psychological adjustment (Storm & Rothmann, 2003; van Vianen, Klehe, Koen, & Dries, 2012). Moreover, such individuals are more likely to appraise stressful events as threats rather than a challenge (Gallagher, 1990). Previous research by Furnham, Crump, and Whelan (1997) has also reported about a strong negative association between neuroticism (low emotional stability) and resilience (Friborg, Barlaug, Martinussen, Rosenvinge, & Hjemdal, 2005). On the other hand, individuals high on emotional stability display effective coping mechanisms and remain calm and less worried (Ang, Van Dyne, & Koh, 2006; Penley & Tomaka, 2002). As a result, individuals with high emotional stability are better able to handle novel situations more effectively, and respond to uncertainty with a greater patience. Furthermore, such individuals display flexible verbal and nonverbal behaviors (Ang et al., 2006) while dealing with others. We, therefore, expect that emotional stability would be strongly related to career resilience.

H1: Emotional stability personality disposition will be strongly related to career resilience.

### Emotional Stability and Psychosocial Mentoring

Past research has depicted that people, who are low on emotional stability are anxious, carry negative emotions because of which they do not show greater interest in initiating mentoring relationships (Bozionelos, 2004). Furthermore, their hostile and impatient attitude also makes them less concerned and less cooperative in understanding the actual needs. Such kind of employees is not able to realize and reap the actual benefits, which can be obtained through the psychosocial support of the mentor. On the other hand, those employees, who are high on emotional stability being calm and composed, are able to regulate their negative emotions. They refrain themselves from being impulsive and do not take things personally, which further enhances the prospects of obtaining mentorship support. Mentors also find it easier to work with those protégés, who are calm and predictable (Lee et al., 2000). We, thus, propose that

H2: Emotional stability personality disposition will be strongly related to psychosocial mentoring.

### Psychosocial Mentoring and Career Resilience

Numerous studies have suggested that managerial support plays a key role in the employee’s career development (Noe, Noe, & Bachhuber, 1990) and other commitment behaviors (Colarelli & Bishop, 1990). The role of managers is also important for inculcating positive feelings in an individual about one’s career (Allen et al., 2004). According to Noe et al. (1990), employees show high levels of career resilience when manager provides performance feedback, communicates the expectations and discusses thoroughly about career-related issues (Noe et al., 1990). Besides this, psychosocial mentoring functions enhance an individual’s ability and may alleviate work-related stress (Greiman, 2007). We, thus, assume that in dealing with emotional reactions that prevail in the workplace during times of adversities and turbulence, psychosocial mentoring would serve as a potential tool to enhance career resilience of the employees.

We have, thus, hypothesized that:

H3: Psychosocial mentoring support will be strongly related to career resilience

### Mediating Role of Psychosocial Mentoring

The role of personality in mentoring relationships has gained a significant importance in the recent years (Ragins & Kram, 2007; Turban & Lee, 2007). Some of the personality traits being domain specific strongly influence mentoring relationships (Ragins & Kram, 2007). Turban and Dougherty (1994) have identified high internal locus of control, self-monitoring and emotional stability as the main personality traits decisive of a person’s attraction to receive mentoring. Also mentoring represents a dynamic interpersonal relationship between mentor and protégé in which protégé’s personality is highly crucial (Ehrich, Tennent, & Hansford, 2002). We argue that the relationship between emotional stability and career resilience will be mediated by psychosocial mentoring support. This is because psychosocial mentoring support provides the most appropriate route through which protégés feel comfortable to share their problems with mentor on a regular interaction basis (Fagenson-Eland, Marks, & Amendola, 1997). Moreover, *friendly nature and admiration of the mentor* in psychosocial mentoring also contributes to the establishment of interpersonal comfort in the mentor-protégé relationship. This helps in reducing the stress and tension prevalent during the times of turbulence (Kram & Hall, 1989). Similarly, unconditional *acceptance and confirmation* of the mentor also instills confidence in the protégés to tackle the obstacles effectively. Thus, with the mentor assuming various roles of psychosocial mentoring; emotionally stable protégés, who show interest and attempt to initiate mentoring relationships, derive the career benefits associated with mentorship (Ragins & Kram, 2007; Turban & Lee, 2007). This enhances confidence in the protégés to face the tough times effectively, thereby, making them more career resilient. Therefore, it can be hypothesized that:

H4: Psychosocial mentoring will mediate the relationship between emotional stability and career resilience.

## Method

### Participants and Procedure

The data for the study were collected from 233 managers having full time employment in private sector (45.1%) and public sector (54.9%) organizations in North India. Of these 233 participants, about 85.4% were males and 14.6% were females with about 39.1% aged within 26–30 years. Participants were from all the three hierarchical levels, junior level management (24.5%), middle level management (60.5%) and senior level management (15%). Overall participants had a good educational background with 6% as diploma holders, 43.8% as graduates, 46.4% as post graduates and 3.8% held degrees higher than post-graduation.

The organizations were targeted by contacting HR managers. These organizations were located in the same geographical region and had similar kind of organizational structure. None of these organizations had a formal mentoring scheme in place at the time of the study. Survey questionnaires were distributed among the participants during the office hours with the support of human resource department of the organization. The aim of the study was then explained to the participants and those managers, who provided their consent, were then administered with a paper and pencil survey. The survey questionnaires, which were distributed, were prefaced with a cover letter that explained the importance of participation. Participants were also assured regarding the anonymity and confidentiality of their responses. Survey questionnaires were initially administered among 240 respondents, seven of whom responded to be not involved in any current mentoring relationship, which ultimately resulted in overall relatively complete data of 233 participants.

### Measures

#### Emotional stability

For the measurement of emotional stability, ten items were adopted from Goldberg’s (1990) Big Five markers available to use freely for research purpose in the International Personality Item Pool (IPIP). The sample items included, “I get stressed out easily”, “I am relaxed most of the time”. The IPIP scales have been found to be most valid for the Indian sample in comparison to other personality scales in a recent study by Uppal, Mishra, and Vohra (2014). The participants were asked to rate themselves on the response scale ranging from 1, “strongly disagree”, to 5, “strongly agree”. The scale’s alpha reliability was found to be .73.

#### Psychosocial mentoring support

We have measured psychosocial mentoring support using the 14-item scale from the study of Noe (1988). The author reported good internal consistency of .92 for this scale. As the original scale items were developed and tested on the sample of educational administrators; it was important to modify the scale items to suit the organizational context. Examples of the items are “My mentor has demonstrated well listening skills in our conversations” and “My mentor has conveyed feelings of respect for me as an individual”. The response scale ranged from 1, “strongly disagree” to 5, “strongly agree”. The scale demonstrated good internal consistency, Cronbach’s α = .92, for the Indian sample.

#### Having a mentor

This was assessed using a single item. An established definition of mentor was also provided to the participants to help them understand meaning of mentor as given below:

A mentor is generally defined as a higher ranking, influential individual in your work environment who has advanced experience and knowledge and who is committed to provide upward mobility and support to your career. Your mentor may or may not be in your organization and may or may not be your immediate supervisor (Ragins, 1989, p. 2).

Following this, they were also asked to respond whether they currently had a mentor (coded No: 1 and coded Yes: 2) in line with previous studies (Bozionelos & Wang, 2006).

#### Career resilience

To measure the career resilience, four items were adopted from the scale of Career Commitment Measure (CCM) (Carson & Bedeian, 1994). CCM represents a multidimensional construct of three major dimensions—career identity, career resilience and career planning. In this study, we have considered career resilience component of the CCM. Sample items include “Given the problems I encounter in this line of work/career field”, “I sometimes wonder if I get enough out of it”, “The costs associated with my line of work/career field sometimes seem too great”. The response scale ranged from 1, “strongly disagree” to 5, “strongly agree”. The scale’s internal consistency Cronbach’s α was reported to be 0.72.

#### Test of discriminant validity

We also examined the distinctiveness of the constructs (emotional stability, career resilience and psychosocial mentoring) using confirmatory factor analysis with the help of Amos software Version 20 (Arbuckle, 2011). To evaluate the goodness-of-fit of the proposed model, we followed maximum likelihood methods of estimation and also considered both absolute and relative fit indices as per the recommendations of Hair, Black, Babin, Anderson, and Tatham (2005) as (a) the χ^2^ goodness-of-fit statistic; (b) the comparative fit indices (CFI; Bentler, 1990); (c) the goodness of fit indices (GFI); (d) the root mean square error of approximation (RMSEA, Steiger, 1990) and (e) the incremental ﬁt index (IFI; Bollen, 1990). The hypothesized model was also compared with three other alternative models using sequential χ^2^ difference test. As depicted in Table 1, the hypothesized model represented most adequate model fit than other alternative models with fit indices as *χ*^2^ (*N* = 233, *df* = 318) = 413.059; CFI = .958; GFI = .890, RMSEA = .036 [RMSEA should be less than .06 for good models (Hu & Bentler, 1998)] and IFI= .959. Henceforth, the hypothesized model was retained for the subsequent analysis.

**Table 1:** Conﬁrmatory Factor Analysis of Measurement Models: Fit Indices

Models		*χ*^2^	*df*	∆*χ*^2^	∆*df*	GFI	CFI	IFI	RMSEA
Hypothesized Model	Combining Three factors^a^	413.059	318	–	–	.890	.958	.959	.036
Model 1	Combining psychosocial mentoring and career resilience	500.659	334	87.6	16	.868	.927	.928	.046
Model 2	Combining emotional stability and psychosocial mentoring support	657.986	333	244.93	15	.822	.857	.860	.065
Model 3	One factor model	539.175	314	126.116	4	.855	.901	.904	.056

^a^Three factors: Emotional stability, psychosocial mentoring and career resilience.

#### Control variables

Following variables were set as controls in order to control their spurious effects on dependent variable and were coded as given below:

- Age (years): 21–25 (1), 26–30 (2), 31–35 (3), 36–40 (4), 41–45 (5), above 45 (6)
- Gender: Males (1), females (2)
- Marital status: Married (1), unmarried (2)
- Educational level: Diploma holder (1), graduates (2), post-graduates (3), higher than post-graduate (4)
- Hierarchical level: Junior level (1), middle level (2), senior level (3)
- Organization sector: Private sector (1), public sector (2).

### Approach for Data Analyses

Multiple hierarchical regression analysis was used for the testing of both direct and indirect effects. Specifically, for the testing of mediation hypothesis, guidelines of Baron and Kenny‘s mediation approach was adopted in alignment with earlier studies (Karatepe, 2011). This technique is based on the notion that mediation effect of the variable first assumes the presence of total effect between predictor and criterion variable initially rather than indirect effect (Mathieu & Taylor, 2006). And this “mediation should justify such a significant total relationship existing between an antecedent and criterion that is accounted either in part (partial mediation) or complete (full) mediation by the respective mediator variable” (Mathieu & Taylor, 2006). Baron and Kenny (1986) proposed sequential verification of four conditions for the fulfillment of conduction of mediation analysis (Pardo & Román, 2013) as (1) independent variable (IV) should relate to dependent variable (DV); (2) independent variable (IV) should relate to the mediator variable (MV); (3) mediator variable (MV) should relate to the dependent variable (DV), and (4) the relationship between independent variable (IV) and the dependent variable (DV) must be significantly reduced in the presence of mediator variable.

Besides these four conditions, another unique distinction was illustrated in context to condition supporting ‘full mediation’ and ‘partial mediation’. According to them, the condition of full mediation is well established when relationship between IV and DV completely disappears while controlling the effect of MV. On the other hand, the condition of partial mediation is considered to be established when the relationship between IV and DV is significantly reduced while controlling MV, but does not completely disappear (Baron & Kenny, 1986; Pardo & Román, 2013).

## Results

The descriptive statistics and inter-correlations among the study variables have been detailed in Table 2. It can be seen that reliabilities have been represented diagonally and all are within the acceptable range of above .70 (Nunnally, 1978). Table 2 shows that emotional stability has positive, but moderate correlation with career resilience (*r* = .211, *p* < .01); and psychosocial mentoring support also relates positively but has weak correlation with career resilience (*r* = .172, *p* < .01).

**Table 2:** Means, Standard Deviations, and Intercorrelations of the Variables

Variable	*M*	*SD*	1	2	3	4	5	6	7	8	9
1. Age	2.901	1.625	–								
2. Gender	1.145	.353	-.275**	–							
3. Marital Status	1.356	.479	-.563**	.200**	–						
4. Educational Level	2.480	.670	-.281**	.194**	.162*	–					
5. Organization Sector	1.566	.546	.311**	-.184**	-.280**	-.182**	–				
6. Hierarchical Level	1.905	.622	.447**	-.113	-.262**	.047	-.019	–			
7. Psychosocial Mentoring	3.814	.713	.163*	-.051	-.123	.015	.252**	.089	[.92]		
8. Emotional Stability	3.257	.635	.177**	-.155*	-.054	-.122	.194**	.071	.172**	[.73]	
9. Career Resilience	3.035	.826	.074	.089	-.054	-.039	.065	.080	.172**	.211**	[.72]

*Note*. *N* = 233. Cronbach’s alpha is presented in parentheses along the diagonal.

*p < .05. **p < .01.

### Hypotheses Testing

For investigation of the direct effects, multiple hierarchical regression analysis was used as the statistical technique. First of all, direct effect of emotional stability was measured on career resilience. The results are demonstrated in Table 3. As hypothesized, the emotional stability personality factor was found as a significant predictor of career resilience (β = .216, *p* < .01), which supported H1. Second, for the evaluation of H2, direct impact of emotional stability personality dimension on the psychosocial mentoring was measured. Table 3 depicted emotional stability personality factor (β = .127, *p* < .05) as a significant predictor of psychosocial mentoring that provided support to H2. Similarly, psychosocial mentoring support (β = .161, *p* < .05) was also seen as a significant predictor of career resilience (see Table 4). Hence, H3 was also supported.

**Table 3:** Hierarchical Multiple Regression Analysis: Direct Effects Between Emotional Stability and Career Resilience; Emotional Stability and Perceived Psychosocial Mentoring

Predictor variables	Career-Resilience					Psychosocial Mentoring				
	ß (Step 1)	ß (Step 2)	*R*^2^	∆*R*^2^	∆*F*	ß (Step 1)	ß (Step 2)	*R*^2^	∆*R*^2^	∆*F*
Step 1 (Control Variables)			.027	–	1.06			.081	–	3.30**
Age	.033	.006				.086	.070			
Gender	.131	.152*				.009	.021			
Marital Status	-.014	-.035				-.009	-.021			
Educational Level	-.044	-.031				.080	.087			
Organization Sector	.069	.035				.240	.220			
Hierarchical Level	.080	.072				.050	.045			
Step 2			.071	.043**	10.48**			.096	.015*	3.71*
Emotional Stability		.216**					.127*			

*Note*. *N* = 233.

*p < .05. **p < .01.

**Table 4:** Hierarchical Multiple Regression Analysis: The Direct Effect of Psychosocial Mentoring on Career Resilience

Predictor variables	Career-Resilience				
	ß (Step 1)	ß (Step 2)	*R*^2^	∆*R*^2^	∆*F*
Step 1 (Control Variables)			.027	–	1.06
Age	.033	.019			
Gender	.131	.130			
Marital Status	-.014	-.013			
Educational Level	-.044	-.057			
Organization Sector	.069	.030			
Hierarchical Level	.080	.072			
Step 2			.051	.024*	5.67*
Psychosocial Mentoring		.161*			

*Note*. *N* = 233.

*p < .05. ** *p* < .01.

### Mediation Analysis Results

The first three conditions of the four step model as proposed by Baron and Kenny (1986) has been well proved with the testing of the hypothesis pertaining to estimating direct effects as depicted in Table 3 and Table 4. For the evaluation of the fourth condition and the final stage of four step model as per Baron and Kenny‘s (1986) proposal, sequential procedure of hierarchical multiple regression analysis was followed in which control variables were first entered in block 1 in the first step; then in the second step, emotional stability (IV) was entered in block 2; and the last step was completed with the entry of psychosocial mentoring support (MV) in block 3 against career resilience (DV).

Table 5 shows that with the entry of psychosocial mentoring support as the mediator variable (MV), the coefficient of relationship between emotional stability (IV) and career resilience (DV) reduced (β = .216, *p* < .01 to β = .199, *p* < .01), but still remained significant while controlling the effects of MV. This indicates partial mediating effect of psychosocial mentoring support on emotional stability and career resilience relationship. Hence, H4 is partially supported.

**Table 5:** Hierarchical Multiple Regression Analysis: Mediating Effects

Predictor variables	Career Resilience (DV)					
	ß (Step 1)	ß (Step 2)	ß (Step 3)	*R*^2^	∆*R*^2^	∆*F*
Step 1 (Control Variables)				.027	–	1.06
Age	.033	.006	-.004			
Gender	.131	.152*	.149*			
Marital Status	-.014	-.035	-.033			
Educational Level	-.044	-.031	-.043			
Organization Sector	.069	.035	.005			
Hierarchical Level	.080	.072	.066			
Step 2 (IV)				.071	.043**	10.48**
Emotional Stability		.216**	.199**			
Step 3 (MV)				.087	.017*	4.09*
Psychosocial Mentoring			.136*			

*Note*. *N* = 233.

*p < .05. **p < .01.

## Discussion

This study makes useful contribution to the available literature on mentoring and occupational psychology from the perspective of understanding the role of socio-emotional support as a mediator of the relationship between emotional stability personality factor and career resilience. To the best of our knowledge, the present study appears to be the first research to explore mediating role of psychosocial mentoring between the two variables. In addition, this study also focuses on understanding the interrelationships between emotional stability and career resilience, emotional stability and psychosocial mentoring, psychosocial mentoring and career resilience, as not many studies have investigated the influence of personality and mentoring on career resilience. In support of Hypothesis H1, emotional stability personality disposition was found to be a positive significant predictor of career resilience. This implies that those protégés, who exhibit greater self-control, remain calm and composed and regulate their emotions, are higher on career resilience. On the other hand, people with low emotional stability (i.e. high neuroticism) are more vulnerable to emotional distress and exhibit poor coping mechanisms, which further makes them a low scorers on the resilience dimension (Campbell-Sills, Cohan, & Stein, 2006; Costa & McCrae, 1992b; Kling, Ryff, Love, & Essex, 2003).

As proposed, for Hypothesis H2, emotional stability personality was found to be significant predictor of psychosocial mentoring support. This means that protégés with higher scores on emotional stability show greater interest and willingness to receive psychosocial mentoring support and vice-versa. Besides this, such individuals may also perceive greater psychosocial mentoring support in comparison to protégés, who are low on emotional stability. This is because protégés with low emotional stability lack confidence for establishing relationships with upper-level managers (Mortazavi, 2012), and are considered as liability because of their downplaying abilities and successes (Turban & Lee, 2007). Also, being low on emotional stability is likely to delay their progress at all the stages of the mentoring relationship, thereby, resulting in dissatisfaction, and may also give rise to instances of conflict that may take the form of a dysfunctional mentoring (Ozer & Benet-Martinez, 2006; Ragins & Kram, 2007; Turban & Lee, 2007). On the other hand, emotionally stable protégés perceive greater psychosocial mentoring support, and are keen to initiate mentoring relationships. As a result, such people are often seen as more competent and attractive by the mentors due to their inherent quest for learning (Turban & Lee, 2007).

Further evaluation of Hypothesis H3, showed that among Indian managers’ psychosocial mentoring acts as a significant predictor of career resilience, which is similar to the proposed hypothesis. This highlights the subjective nature of psychosocial mentoring in influencing employees’ affective reactions for individual level and organizational level outcomes (Ammeter, Douglas, Gardner, Hochwarter, & Ferris, 2002; Ashkanasy & Daus, 2002; Craig, Allen, Reid, Riemenschneider, & Armstrong, 2013; Weiss & Cropanzano, 1996). Similarly, in support of Hypothesis H4, the findings of our study depicted a partial mediating effect of psychosocial mentoring support on emotional stability and career resilience relationship. Undoubtedly, psychosocial mentoring provides a strong mechanism for linking emotional stability personality disposition and career resilience; yet the finding about partial mediation of psychosocial mentoring shows that for administering mentoring, it is important to consider protégé’s personality traits, specifically, those protégés, who possess the desired traits are more likely to show interest to get the mentoring and extract the key benefits associated with mentoring process. Another reason that can be attributed to the partial mediation effect of psychosocial mentoring is the possible inclusion of other mediator-moderator variables those have not been focused in this research. For example, the recent study by Kao et al. (2014) demonstrated the effects of mentor’s gender and supervisory status on resilience in mentoring relationships. Similarly, as vocational support facilitated by the mentor is also known to influence employee’s resilience (Kao et al., 2014); therefore, one might expect mediation of career-oriented mentoring in addition to psychosocial mentoring support on emotional stability and career resilience relationship.

### Practical Contributions of the Present Study

The results of the study provide meaningful implications from both practical and theoretical perspectives. These are discussed as below:

#### Recruitment and selection

As, emotional stability personality disposition is closely related to “successful functioning” in all areas of life including situations of adversity, both in and out of the workplace (Bailey & Gulko, 2014); this also represents a major call for organizational decision-makers to be alert during recruitment and selection to focus on only those candidates, who are high on emotional stability. Moreover, people, who are high on emotional stability are rational decision-makers and are less judgmental in their behavior; therefore, bringing in emotionally stable candidates will serve as a step forward to sound development of workplace interpersonal connections and in maintaining cordial-friendly relations (Rajasekher, 2011).

#### Grounding psychosocial mentoring skills

Given the crucial role of protégé’s positive perceptions of psychosocial mentoring in the development of positive attitudes, such as organizational commitment, organizational attractiveness and intentions to pursue employment in the mentor’s organizations (Spitzmüller et al., 2008; Windeler & Riemenschneider, 2013); we also suggest policy-makers of the organization to take initiatives for inculcating psychosocial mentoring skills in the potential mentors of the organization. This could be done through organizing sessions, like Training-for-Trainers to help them understand the usefulness of psychosocial mentoring roles. Particularly, managers must be trained for how they should provide negative feedback to employees in a constructive manner without unduly criticizing them (Willemyns, 2010); this is especially most required while dealing with protégés, who are low on emotional stability.

#### Counseling practices

Specifically, the findings of this research have implications for incorporating counseling practice in the mentorship model of the organizations so as to guide the workforce during the times of turbulence. For example, if a protégé is low on emotional stability, his anxiousness and negative affectivity can be reduced to some extent with mentor’s counseling skills. As counseling helps in assessing the degree of anxiety and depression that may influence the client’s decision-making ability; additionally, it may also help protégés in overcoming challenges of their multiple roles by enhancing their self-efficacy and self-confidence (Perrone & Civiletto, 2004; Salami, 2008).

#### Designing mentoring programs

The findings of the study might be useful for preparing guidelines for designing mentoring programs in the organization. One of the noteworthy, implications of this research is that it informs both mentors and protégés about how important it is to maintain friendly and unconditional support in the mentor-protégé relationship in order to achieve outcomes in the long run.

### Limitations and Recommendations for Future Research

There are some limitations associated with the present research. First limitation is the use of a cross-sectional survey based research design, which is based on protégé’s self-reported data that limits the scope for making any inferences about causality. For instance, our study revealed psychosocial mentoring as the significant predictor of career resilience. Yet, one might think of a career-resilient protégé to perceive higher levels of psychosocial mentoring support. Perhaps, such kind of causal inferences could be determined through the adoption of longitudinal research designs.

Second concern is related to the common-method bias (CMB) due to the use of self-reported measures in the study. However, this does not represent serious threat to our study, as checked by using the procedural guideline of Harman’s single factor (Podsakoff & Organ, 1986), which showed the emergence of a single factor that explained very little variance.

Further, as scope of the study was limited to study the linkage between psychosocial mentoring, emotional stability and career resilience; sample inclusion took into consideration only the mentored individuals. Further study is recommended using the experimental and quasi-experimental research designs while considering both mentored as well as non-mentored individuals.

As consent prescreening is considered as one of the promising techniques for increasing response rates (Baruch & Holtom, 2008); the data of this study were collected through personal survey administration after the managers expressed their consent which has resulted in high response rates. This may be questioned due to general association of high R-R with the mixed-mode approach of surveys (Shih & Fan, 2007). Future studies are, thus, recommended to incorporate mixed-mode methods for conducting survey research.

Another concern is the sample intake from different industries; hence, findings cannot be generalized to any specific sector. Future studies are recommended for testing the proposed interrelationships in the specific business setting. In this regard, scholars and academicians may seek to extend the work by exploring various probable subsets of psychosocial mentoring functions in a specific organizational context. Also, given the shortcomings in the development of a psychosocial mentoring relationship in organizations with formal mentorship scheme, a qualitative research investigation may be of interest for preparing comparative account of the formal mentoring versus informal mentoring.
